# Bioenergetic changes in response to sperm capacitation and two-way metabolic compensation in a new murine model

**DOI:** 10.1007/s00018-022-04652-0

**Published:** 2022-12-19

**Authors:** Maximiliano Tourmente, Ester Sansegundo, Eduardo Rial, Eduardo R. S. Roldan

**Affiliations:** 1 Department of Biodiversity and Evolutionary Biology, Museo Nacional de Ciencias Naturales, CSIC, Madrid, Spain; 2 Centro de Biología Celular y Molecular, Facultad de Ciencias Exactas, Físicas y Naturales (FCEFyN - UNC), Universidad Nacional de Córdoba, Córdoba, Argentina; 3 Instituto de Investigaciones Biológicas y Tecnológicas, Consejo Nacional de Investigaciones Científicas y Técnicas (IIByT - CONICET, UNC), Córdoba, Argentina; 4 Department of Structural and Chemical Biology, Centro de Investigaciones Biológicas Margarita Salas, CSIC, Madrid, Spain

**Keywords:** Metabolism, Sperm, Capacitation, Metabolic compensation, *Mus spicilegus*

## Abstract

The acquisition of fertilizing ability by mammalian spermatozoa, known as “capacitation,” includes processes that depend on particular metabolic pathways. This has led to the hypothesis that ATP demands might differ between capacitated and non-capacitated cells. Mouse sperm can produce ATP via OXPHOS and aerobic glycolysis, an advantageous characteristic considering that these cells have to function in the complex and variable environment of the female reproductive tract. Nonetheless, despite evidence showing that both metabolic pathways play a role in events associated with mouse sperm capacitation, there is contradictory evidence regarding changes promoted by capacitation in this species. In addition, the vast majority of studies regarding murine sperm metabolism use *Mus musculus* laboratory strains as model, thus neglecting the wide diversity of sperm traits of other species of *Mus*. Focus on closely related species with distinct evolutionary histories, which may be the result of different selective pressures, could shed light on diversity of metabolic processes. Here, we analyzed variations in sperm bioenergetics associated with capacitation in spermatozoa of the steppe mouse, *Mus spicilegus*, a species with high sperm performance. Furthermore, we compared sperm metabolic traits of this species with similar traits previously characterized in *M. musculus*. We found that the metabolism of *M. spicilegus* sperm responded to capacitation in a manner similar to that of *M. musculus* sperm. However, *M. spicilegus* sperm showed distinct metabolic features, including the ability to perform cross-pathway metabolic compensation in response to either respiratory or glycolytic inhibition, thus revealing a delicate fine-tuning of its metabolic capacities.

**Supplementary Information:**

The online version contains supplementary material available at 10.1007/s00018-022-04652-0.

## Introduction

In mammals, the ATP used to maintain flagellar beating and, hence, cell motility is produced through two distinct metabolic pathways, oxidative phosphorylation (OXPHOS) and aerobic glycolysis [1–3]. These processes are spatially separated due to the highly polarized and compartmentalized morphology of the spermatozoon. OXPHOS occurs in the mitochondria that are circumscribed to the midpiece while most glycolytic enzymes are anchored to the fibrous sheath, along the principal piece of the flagellum [1–4]. Although the relative importance of each ATP production pathway varies widely across mammalian species [4], the metabolic requirements of the sperm also vary in response to post-ejaculatory physiological changes [5].

The availability of metabolic substrates varies between the different regions of the female reproductive tract and is also subjected to changes along the female reproductive cycle [6–9]. In this context, the ability of sperm to adjust their metabolism to metabolic substrate availability would be advantageous from an evolutionary point of view. Moreover, this phenomenon has been observed in other cell types that face highly variable environments during their lives [10, 11].

Mammalian sperm are unable to fertilize the egg immediately after ejaculation and must acquire fertilizing ability through a series of structural, biochemical, physiological, and behavioral modifications, collectively known as capacitation [12–16]. Sperm capacitation is a cell encompassing process that includes alterations in the composition and structure of the plasma membrane [17–19], changes in intracellular ionic composition [20–22], activation of lipid signaling pathways involving phospholipase A_2_ [23], activation of the cAMP-dependent PKA pathway [24–26], and pervasive tyrosine and serine-threonine phosphorylation [27–29]. These changes endow the sperm with the ability to guide their movement toward the oocyte through chemotaxis [30, 31] and acquire a highly vigorous and asymmetrical pattern of flagellar beating, a process known as “hyperactivation” [32–34]. Furthermore, these modifications lead to the exocytosis of the acrosomal granule [35, 36], which allows sperm to penetrate the oocyte vestments and fuse with the oolema. Sperm metabolism is fundamental for the normal occurrence of capacitation since many of the events associated with this process depend on the activity of particular metabolic pathways in a species-specific manner [5]. This leads to the hypothesis that some of the physiological changes that take place during this process (e.g., counter-gradient ion transport, extensive protein phosphorylation, and hyperactivation) would affect the energy demand at cellular or subcellular levels [37, 38].

Mouse sperm use OXPHOS and glycolysis as energy-producing pathways [39] and appear capable of partially compensating a decrease in respiratory function by increasing glycolytic rate for a short period [40]. These features would favor sperm performance inside the chemically variable reproductive tract of female mice [41, 42]. However, the loss of function of any of the two main metabolic pathways for a protracted period leads to pronounced alterations in their normal flagellar motility [43–47]. Moreover, there is substantial evidence showing that glycolysis is fundamental for the correct development of many events in the capacitation process [37, 48–52], and recent studies have revealed that the maintenance of mitochondrial membrane potential plays a key role in hyperactivation of mouse sperm [53, 54].

The evidence regarding how capacitation affects changes in mouse sperm bioenergetics is somewhat contradictory. It has been reported that pharmacologically induced capacitation causes an elevation in glucose consumption, which was associated with an increase in the rates of both respiration and glycolysis during capacitation time [55]. However, we have recently found that the energy metabolism of these cells shifts from oxidative to glycolytic after incubation under capacitating conditions [40].

The vast majority of evidence regarding murine sperm metabolism corresponds to studies on strains of the laboratory mouse (*Mus musculus domesticus*). Nonetheless, there is a wide range of variation in sperm motility and bioenergetic performance between mouse species, which represents an opportunity to dissect metabolic pathways in different physiological scenarios. A study using extracellular flux analyses to compare sperm metabolism between the house mouse and other species of the genus *Mus* revealed that the ratio of usage of each metabolic pathway varied in a species-specific manner [39]. Further studies showed that the species with more oxidative metabolism [39] were able to sustain higher rates of ATP synthesis [56] to fuel faster swimming velocities for longer periods [57]. To the best of our knowledge, the association between capacitation and energy metabolism in *Mus* species has only been compared in one study [58], which showed that two species (*M. spretus* and *M. spicilegus*) experienced a more severe decline in sperm ATP concentration, as a consequence of capacitation, than *M. musculus*.

In the present study, we assessed the variations promoted by capacitation in the energy-producing metabolism of *M. spicilegus* sperm by using extracellular flux analysis (hereafter EFA). Furthermore, we compared these bioenergetic parameters with the results shown for *M. musculus* in a previous study [40]. We conclude that capacitation promotes a change in *M. spicilegus* sperm toward a more glycolytic metabolism, similar to what occurs in *M. musculus*. On the other hand, the sperm of *M. spicilegus* exhibits particular metabolic features, such as greater metabolic flexibility, which may be regarded as adaptations to more intense levels of performance required to ensure fertilization in competitive situations.

## Materials and methods

### Reagents

Unless otherwise stated, reagents were acquired from Merck (Madrid, Spain).

### Experimental animals, sperm collection, and incubations

Adult male *Mus spicilegus* (3–5 months old) were obtained from the Institut des Sciences de l’Évolution-Montpellier, CNRS-Université Montpellier 2, France. The mice were kept at the animal facilities of the Museo Nacional de Ciencias Naturales (Madrid, Spain) in individual cages at 20–24 °C with a 14 h light and 10 h darkness photoperiod. Animals had water and food available ad libitum.

Mice were dissected after sacrifice and both caudae epididymides were extracted. After removing blood vessels, fat and surrounding connective tissue, each cauda epididymis was placed in a 35-mm Petri dish containing 1 ml of one of two variants of culture medium at 37 °C. One cauda epididymis was placed in non-capacitating medium under air, and the other one was placed in capacitating medium under 5% CO_2_/air. The composition of both incubation media was based on a Hepes-buffered modified Tyrode’s medium [59], that was supplemented with albumin, lactate, and pyruvate (pH = 7.4, osmolality = 295 mOsm kg^−1^). The composition of the non-capacitating medium was: 132 mM NaCl, 2.68 mM KCl, 0.49 mM MgCl_2_.6H_2_O, 0.36 mM NaH_2_PO_4_.2H_2_O, 5.56 mM glucose, 20 mM Hepes, 1.80 mM CaCl_2_, 0.02 mM phenol red, 0.09 mM kanamycin, 4 mg ml^−1^ fatty acid-free BSA, 20 mM Na lactate, and 0.5 mM Na pyruvate. The capacitating medium had an equivalent formulation, but 15 mM NaCl were replaced with 15 mM NaHCO_3_ to maintain osmolarity.

Sperm were collected by performing three to five incisions in the distal region of the caudae and allowing the cells to swim out for 5 min [58]. After swim-out, the epididymal tissue was discarded and the sperm suspension was placed in a plastic tube under a suitable atmosphere. Sperm from two mice were pooled for each extracellular flux analysis (EFA) experiment (the two sperm suspensions in each condition mixed in one incubation tube). Sperm concentrations were adjusted to 100 × 10^6^ sperm ml^−1^ in each suspension after estimating concentration with a modified Neubauer chamber. Sperm suspensions were then incubated for 1 h at 37 °C under the corresponding atmosphere for each medium. This time of incubation was selected since *M. spicilegus* sperm populations achieve the peak percentage of capacitated cells (~ 70%) at this time [58]. Large-bore pipette tips were used in all procedures to minimize damage to spermatozoa. One male per experiment was used for the assessment of ATP content. In this case, sperm were incubated for 1 h at a concentration of 20 × 10^6^ sperm ml^−1^ prior to measurements.

### Ethics approval statement

The animals used in this study were cared and maintained according to the guidelines of European Union Regulation 2010/63 and the Royal Decree for the Protection of Experimental Animals RD53/2013; animal handling was performed with the approval of CSIC's ethics committee and the Comunidad de Madrid (28079-47-A). Animals were sacrificed by cervical dislocation. No other procedures were included in this study.

### Evaluation of OXPHOS and glycolysis rates

Oxygen consumption rate (OCR) and extracellular acidification rate (ECAR) were measured in real-time using an XF24 extracellular flux analyzer (Agilent Seahorse, Santa Clara, CA). OCR indicates the level of respiratory activity in a population of cells, and ECAR, which is proportional to the rate of lactate excretion, is used as a proxy for the rate of aerobic glycolysis. The method used in this study to assess OCR and ECAR follows the original technique developed by Tourmente et al. [39], with minor modifications.

Before the experiment, 30 µl of 0.2 mg/ml natural mouse laminin (Invitrogen, Madrid) in PBS were added to each well of a 24-well XF24 plastic microplate and incubated for at least 3 h at room temperature. Before adding cells to the wells, the excess laminin was rinsed with 1 ml of ultrapure water and discarded. After the 1-h incubation in either non-capacitating or capacitating medium, sperm were transferred to the laminin-coated plate. Ten wells were seeded with 100 µl of sperm suspension (approximately 10 × 10^6^ sperm) that were previously incubated under non-capacitating conditions, other 10 wells were seeded with sperm suspension incubated under capacitating conditions, and four wells were left without cells to perform background corrections. After 3 min, the plate was centrifuged for 1 min at 1200 × *g* in each direction to ensure an even distribution of attached cells throughout the bottom of the well. The incubation medium was then discarded from each well and was immediately replaced with 500 µl of assay medium. Since the assessment of ECAR is only possible in a medium with low pH buffering capacity, an unbuffered assay medium was used for the measurements and dilution of the metabolic modulators. In this medium, Hepes and NaHCO_3_ were replaced with NaCl to preserve osmolality, and pH was adjusted to 7.4 at 37 °C. Finally, the plate with the sperm was placed in the XF24 apparatus, and a 12-min equilibration step was allowed before assessments. The use of laminin as sperm adhesive, instead of the concanavalin A used in the original protocol [39], produced a more uniform and continuous layer of adhered cells (Fig. S1). Moreover, while concanavalin A adhered sperm in a nonspecific manner (i.e., either by the head or the flagellum), sperm were attached to the laminin coating only by their heads while their flagella were beating freely in the medium.

In each experiment, OCR and ECAR were recorded during 54 min (9 cycles of 6 min, consisting of 3 min of measurement, 2 min of mixing, and 1 min of waiting). During the first four cycles, sperm metabolic rates were measured without any additions. Subsequently, cells in each well were treated with either assay medium (baseline conditions), or one of four metabolically active compounds via automatic injection, and sperm metabolism was recorded for 3 additional cycles. Metabolic modulators were: (a) 5 μM oligomycin A, an inhibitor of the mitochondrial ATP synthase, (b) 1 µM carbonyl cyanide p-trifluoro-methoxyphenylhydrazone (FCCP), an uncoupler of mitochondrial respiration, (c) 50 mM 2-deoxy-d-glucose (2DOG), a glucose analog that competitively inhibits the first step of glycolysis, and (d) 30 mM sodium oxamate, a competitive inhibitor of the enzyme lactate dehydrogenase (LDH). Finally, 1 µM rotenone and 1 µM antimycin A were added to the wells and two final measurements were performed to complete a total experiment duration of 54 min. Oxygen levels were able to fully recover after the marked descent occurring during the measurement (Fig. S2), indicating that the sperm concentration used in the experiment was not enough to cause hypoxia. Cells were treated with oligomycin in four separate experiments, and with 2DOG, oxamate and FCCP in three experiments since the large number of wells made possible the application of more than one treatment in each experiment. The first measurement was discarded in all experiments since, according to our previous experience, it tends to show a higher degree of instability.

After the end of the experiment, the sperm in each well were resuspended by scratching the bottom of the well with a pipette tip under a phase-contrast microscope at 10 × , and 10 µl of sperm suspension were collected to estimate sperm concentration in each well. OCR and ECAR values for each well were normalized by the number of sperm present and reported as amol of O_2_ min^−1^ sperm^−1^, and nano-pH min^−1^ sperm^−1^, respectively.

### Calculation of metabolic parameters

The assessment of OCR and ECAR on sperm treated with metabolic modulators led to the estimation of metabolic parameters (Table 1) that are more informative than the raw normalized measurements, allowing for a more accurate comparison between the two incubation conditions [60, 61]. Metabolic parameters were calculated for each well as the average value of the measurements taken for that well in condition A, minus the average value of the measurements taken for that well in condition B (see Table S1 for a description of the conditions and Fig. S3 for a graphical representation). Values corresponding to baseline levels (no additions) were averaged across measurement cycles 2–4, and values corresponding to oligomycin (for OCR), 2DOG, and oxamate additions were averaged across cycles 5–7. Exceptionally, in measures of OCR for cells treated with FCCP, and ECAR for cells treated with oligomycin, the highest value among cycles 5–7 was used for calculation instead of the average.

In order to facilitate comparisons with previous studies, we calculated the response of OCR and ECAR to metabolic modulators as a percentage of the averaged basal levels (measures 2–4) for each well. For OCR measurements, the basal and stimulated/inhibited values were first corrected by subtracting the average of measures after the addition of A + R (cycles 8–9).

### Sperm ATP content

Sperm ATP content was compared between sperm incubated in non-capacitating or capacitating conditions (*N* = 3). A luciferase-based ATP assay kit (Roche ATP Bioluminescence Assay Kit HS II) was used, based on the protocol of Tourmente et al. [56]. After 60 min of incubation in either condition, a 100 μl aliquot of diluted sperm suspension was mixed with 100 μl of Cell Lysis Reagent, incubated at room temperature for 5 min, and centrifuged at 12,000 × *g* for 2 min. The supernatant was recovered and frozen in liquid N_2_. The bioluminescent signal was measured in triplicate in 96-well plates using a luminometer (Biotek Synergy, Biotek Instruments Inc.). 50 μl of Luciferase reagent was added to 50 μl of sample in each well via automatic injection, and, following a 1 s delay, light emission was measured over a 5-s integration period. The ATP content for each sample was estimated using standard curves constructed for each plate with solutions containing known concentrations of ATP. ATP content per well was normalized by the number of cells represented in 50 μl of lysate and expressed as amol sperm^−1^.

### Interspecific comparison

The results from the metabolic measurements in the present study were compared with those of a similar study performed recently in the laboratory mouse (*Mus musculus*; B6D2F1) [40]. Although for *M. musculus* metabolic data were also collected using extracellular flux analysis (EFA), differences between studies in the measuring equipment (Agilent Seahorse XFp vs XF24) and the state in which the cells were assessed (free-swimming vs attached sperm) preclude direct comparisons between absolute OCR and ECAR values. Thus, variations in the OCR/ECAR ratio as a consequence of sperm capacitation, and the response of OCR and ECAR to metabolic modulators (oligomycin, and 2DOG) in non-capacitated sperm, were compared between species. The effect of the metabolic modulators on OCR and ECAR was expressed as percentages relative to their baseline levels (without any metabolic modulator). The effect of oligomycin and 2DOG on OCR was calculated as the average across cycles 5–7 for each well, while the effect of these modulators on ECAR was represented by the highest (oligomycin) and lowest (2DOG) values among cycles 5–7 for each well.

### Statistical analyses

All analyses were conducted using R version 4.2.1 (R Foundation for Statistical Computing, Vienna, Austria), with *α* = 0.05. The effects of incubation conditions and metabolic modulators on OCR and ECAR of *M. spicilegus* sperm, expressed as percentages relative to the baseline level, were assessed via mixed effect models (MM, package *afex*, function *mixed*) using incubation condition medium (non-capacitating and capacitating) and treatment (baseline, oligomycin, FCCP, 2DOG, oxamate, and A + R) as fixed factors, and experiment as a random factor. The values of the metabolic parameters calculated after EFAs were compared between incubation conditions using MMs with incubation medium as a fixed factor and experiment as a random factor. The inclusion of the experiment as a random factor allowed for the consideration of each well as an individual value in the statistical analyses, thus decreasing the impact of outliers and increasing the statistical powers of the tests by controlling for the between-experiment variability [62].

The interspecific comparison of the OCR/ECAR ratio and the response of metabolic rates to metabolic modulators was also carried out by means of MMs. In the first case, OCR/ECAR was used as a dependent variable and species and incubation conditions as fixed factors. In the second case, the changes in OCR and ECAR after treatment with oligomycin, and 2DOG (expressed as the percentage relative to the baseline) were used as dependent variables and species as a fixed factor. In the two cases, the experiment was used as a random factor.

Each analysis used either a linear mixed effect model (LMM) with log_10_-transformation applied to the response variable, or a generalized linear mixed effect model (GLMM) with gamma distribution and inverse link function. The type of analysis chosen in each case was the one that produced residuals that better fit the model assumptions. Significant differences between levels of fixed factors were analyzed via *post-hoc* estimated marginal means tests (package *emmeans*, function *pairwise*).

## Results

**Fig. 1:**
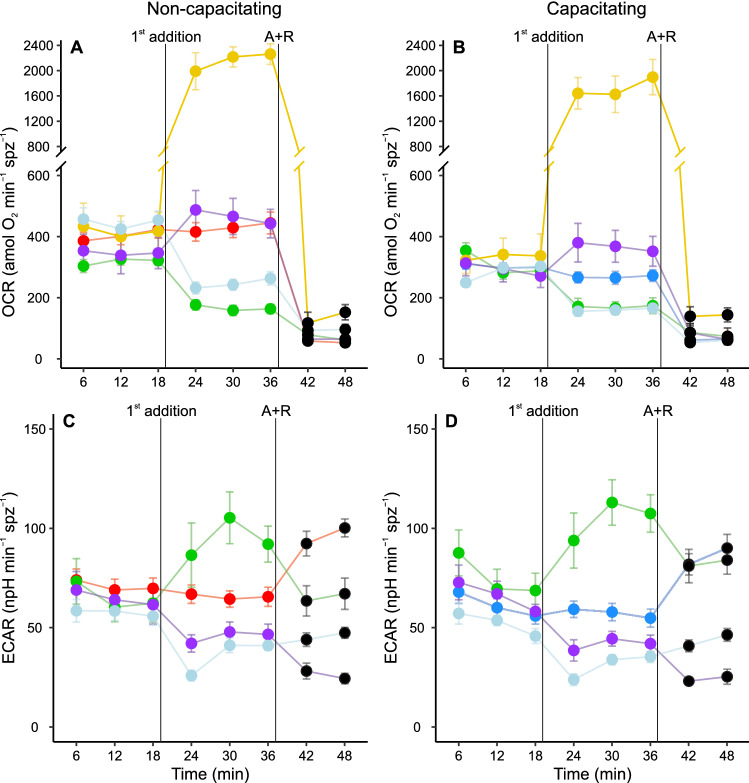
Real-time measurement of the metabolic rates of *M. spicilegus* sperm, preincubated under non-capacitating or capacitating conditions, before and after treatment with metabolic modulators. **A**, **B** Oxygen consumption rate (OCR). **C**, **D** Extracellular acidification rate (ECAR). Sperm were incubated for 1 h in non-capacitating (**A**, **C**) and capacitating (**B**, **D**) medium before EFA. Values have been normalized by sperm numbers inside each well. Symbols and whiskers represent means ± standard error. Time = 0 was defined as the start of the first measurement cycle; measurement cycles 2–9 are reported. The first vertical line (1st addition) marks the addition of either registry medium (basal state), 5 µM oligomycin, 1 µM FCCP, 50 mM 2DOG, or 30 mM sodium oxamate; the line labeled “A + R” marks the addition of 1 µM antimycin A + 1 µM rotenone. Red and blue symbols: basal state (no modulators) in non-capacitating and capacitating conditions, respectively; green symbols: oligomycin treatment; yellow symbols: FCCP treatment; purple symbols: 2DOG treatment; grey symbols: oxamate treatment; black symbols: all treatments after A + R addition

**Fig. 2:**
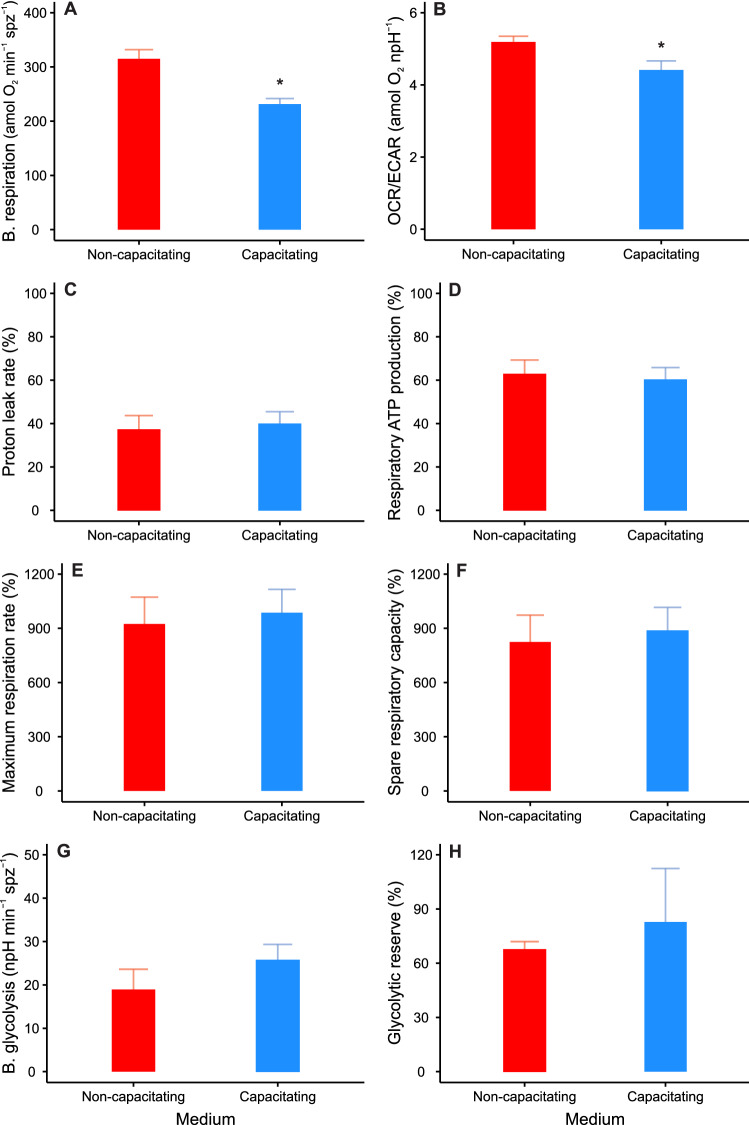
Metabolic parameters and OCR/ECAR ratio of *M. spicilegus* sperm incubated under non-capacitating (red bars) and capacitating (blue bars) conditions. Basal respiration (**A**) and glycolytic (**G**) rates are expressed as per-sperm normalized values. Proton leak (**C**), respiratory ATP production (**D**), maximum respiration rate (**E**), and spare respiratory capacity (**F**) are expressed as percentages relative to the basal respiration rate. Glycolytic reserve (**H**) is expressed as a percentage relative to the basal glycolytic rate. The OCR/ECAR ratio (**B**) was calculated before any addition (basal state). Sperm were incubated in different media (non-capacitating vs capacitating) for 1 h before extracellular flux analysis. Bars represent means + standard error. Asterisks indicate significant differences (*p* < 0.05) in values between incubation media in MMs using incubation conditions as fixed factor and experiment as random factor. Basal respiration and glycolytic rates, and OCR/ECAR were analyzed using LMMs after log_10_ transformation of the response variable. Proton leak, respiratory ATP production, maximum respiration rate, spare respiratory capacity, and glycolytic reserve were analyzed using GLMMs (gamma distribution, inverse link function)

The full profiles for OCR and ECAR measurements in *M. spicilegus* sperm are presented in Fig. 1. Regardless of their preincubation conditions (non-capacitating or capacitating), sperm populations under basal conditions (prior to the addition of the metabolic modulators) showed stable OCR (Fig. 1A, B) and ECAR (Fig. 1C, D) values throughout the experiments. Additionally, basal OCR was significantly decreased after preincubation under capacitating conditions (Table 1; Fig. 2A) while basal ECAR showed a non-significant increment in sperm populations enriched in capacitated sperm (Table 1; Fig. 2G). It should be pointed out that this ECAR increase is underestimated since the contribution of HCO_3_^−^ to the acidification is decreased because the OXPHOS-derived CO_2_ production is lower [63]. In agreement with these results, sperm incubated in non-capacitating medium exhibited a higher basal OCR/ECAR than that of sperm incubated in non-capacitated conditions (Table 1; Fig. 2B). According to this evidence, sperm capacitation would elicit a variation in the relative importance of the two main metabolic pathways (OXPHOS and glycolysis) in *M. spicilegus*. The sperm ATP content was lower in sperm preincubated in capacitating medium (Table 1).

**Table 1:** Effect of incubation conditions on the metabolic parameters, OCR/ECAR ratio, and ATP levels of *M. spicilegus* sperm

Dependent variable	*N*	Non-capacitating			Capacitating			*Χ*^*2*^	*p*
		Mean	SD	CI	Mean	SD	CI		
Basal respiration (amol O_2_ min^−1^ spz^−1^)	6	315	122	34	231	71	21	15.65^a^	** < 0.001**
Proton leak (%)	4	37.2	11.2	7.1	39.8	9.8	6.2	0.54^b^	0.464
Respiratory ATP production (%)	4	62.8	11.2	7.1	60.2	9.8	6.2	0.69^b^	0.407
Maximal respiration (%)	3	924	364	382	987	316	331	0.25^b^	0.615
Spare respiratory capacity (%)	3	824	364	382	887	316	331	0.24^b^	0.622
Basal glycolysis (npH min^−1^)	3	18.9	13.2	11.1	25.7	9.6	8.9	2.16^a^	0.142
Glycolytic reserve (%)	3	67.6	15.1	9.6	82.8	88.7	68.2	1.38^b^	0.240
OCR/ECAR (amol O_2_ npH^−1^)	6	5.19	1.9	0.3	4.42	2.8	0.49	12.84^a^	** < 0.001**
ATP (amol sperm^−1^)	3	124	24	60	70	17	44	11.19^b^	** < 0.001**

Basal respiration and glycolytic rates, and ATP levels are expressed as per-sperm normalized values. Proton leak, respiratory ATP production, maximum respiration rate, and spare respiratory capacity are expressed as percentages relative to the basal respiration rate. Glycolytic reserve is expressed as a percentage relative to the basal glycolytic rate. The OCR/ECAR ratio was calculated before any addition (basal state). *N*: number of independent experiments. Mean values, standard deviations (SD), and 95% confidence intervals of means (CI) are presented. *Χ*^*2*^ and *p* values were estimated by MMs using incubation conditions as fixed factor and experiment as random factor, and likelihood ratio tests. Significant differences (*p* < 0.05) are shown in boldface

^a^Estimated using LMMs after log_10_ transformation of the response variable

^b^Estimated using GLMMs (gamma distribution, inverse link function)

**Fig. 3:**
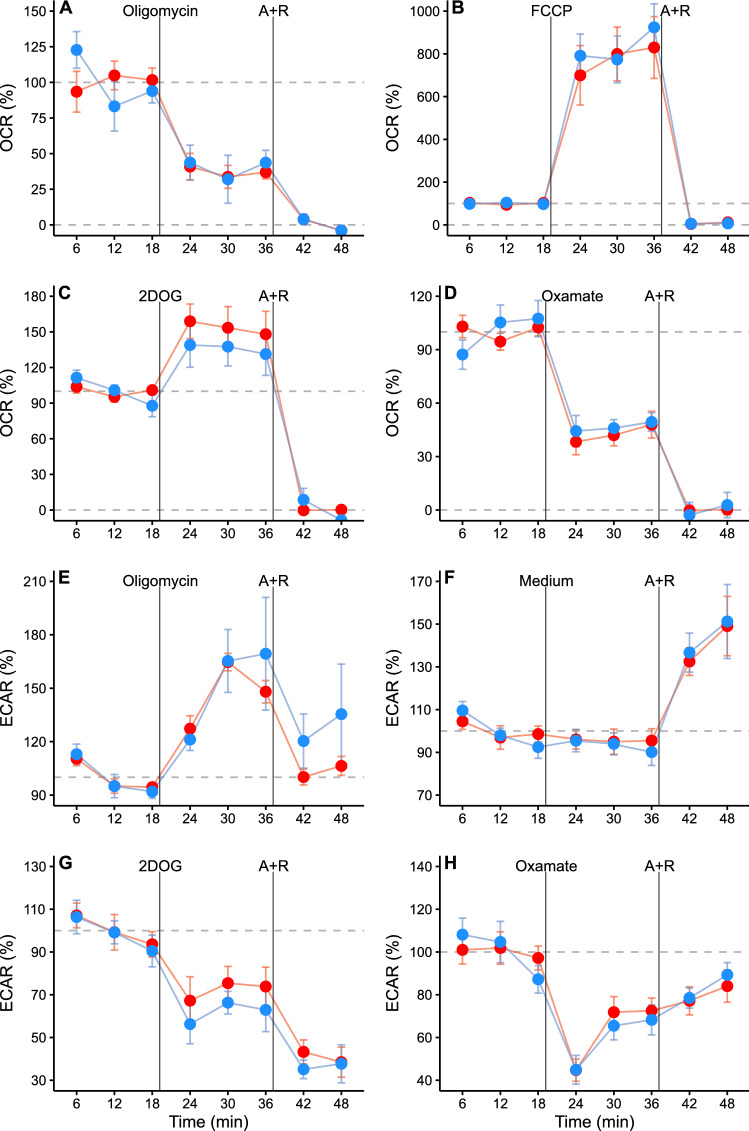
Changes in the metabolic rates of *M. spicilegus* sperm after the addition of metabolic modulators. **A**, **B**, **C**, **D** Oxygen consumption rate (OCR). **E**, **F**, **G**, **H** Extracellular acidification rate (ECAR). Sperm were incubated for 1 h in non-capacitating (red symbols) and capacitating (blue symbols) medium before extracellular flux analysis. Values are expressed as percentages relative to the baseline (average of the three measurement cycles before any addition). Symbols and whiskers represent means ± standard error. Dashed grey lines indicate 100% and 0% levels. Measurement cycles 2–11 are reported. The first vertical line marks the addition of either registry medium, 5 µM oligomycin, 1 µM FCCP, 50 mM 2DOG, or 30 mM sodium oxamate; the line labeled “A + R” marks the addition of 1 µM antimycin A + 1 µM rotenone

**Fig. 4:**
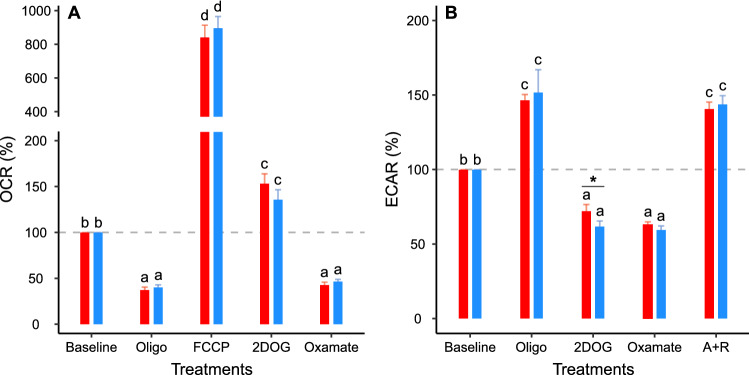
Effect of metabolic modulators and incubation conditions on metabolic rates in the sperm of *M. spicilegus*. **A** Oxygen consumption rate (OCR). **B** Extracellular acidification rate (ECAR). Sperm were incubated for 1 h in non-capacitating (red bars) and capacitating (blue bars) medium before extracellular flux analysis. Values are expressed as percentages relative to the basal rates (100% level indicated by the horizontal dashed line). Bars represent means + standard error. Treatments represent sperm metabolic rates before any addition (baseline) or after the addition of 5 µM oligomycin (Oligo); 1 µM FCCP (FCCP); 50 mM 2DOG (2DOG); 30 mM sodium oxamate (Oxamate); or 1 µM antimycin A + 1 µM rotenone (A + R). Dashed grey lines indicate 100% level. Effects on OCR and ECAR were analyzed using GLMMs (gamma distribution, inverse link function) with treatment and incubation conditions as fixed factors and experiment as random factor. Different letters indicate significant differences (*p* < 0.05) between treatments for the same incubation condition in a post-hoc marginal means test. Asterisks indicate significant differences (*p* < 0.05) between incubation conditions for the same treatment in a post-hoc marginal means test

Sperm treatment with metabolic modulators provoked marked variations in OCR and ECAR (Table 2; Figs. 1, 3) that allowed a detailed characterization of the energy metabolism and revealed a number of *M. spicilegus* specific cross-pathway effects. The magnitude of the responses relative to the baseline values was statistically similar between preincubation conditions (Table 2; Fig. 4A, B). A notable exception to this general trend was the effect of 2DOG in ECAR, which was slightly more pronounced in capacitating sperm. This was confirmed by the significant interaction term in the corresponding statistical analysis (Table 2, Fig. 4B).

**Table 2:** Effect of incubation conditions and metabolic modulators on *Mus spicilegus* sperm OCR and ECAR

Dependent variable	*N*	Treatment	Non-capacitating			Capacitating			Independent variable	*X*^*2*^	*p*
			Mean	SD	CI	Mean	SD	CI			
OCR (%)	4	Oligomycin	37.3	11.2	7.12	40.19	9.66	6.13	Incubation medium	2.01	0.157
									Treatment	652.62	** < 0.001**
	3	FCCP	841	357	374	896	339	356	Interaction	6.02	0.197
	3	2DOG	153	30.5	25.5	136	26.7	28.0			
	3	Oxamate	42.7	9.47	7.28	46.6	7.35	5.65			
ECAR (%)	4	Oligomycin	147	13.7	8.68	152	45.9	35.3	Incubation medium	5.06	**0.025**
									Treatment	363.56	** < 0.001**
	3	2DOG	72.1	12.7	10.6	61.8	9.93	9.19	Interaction	9.63	**0.047**
	3	Oxamate	63.0	5.83	4.48	59.5	7.54	6.31			
	6	A + R	141	21.9	9.72	144	27.5	12.2			

Both metabolic variables are expressed as percentages relative to the baseline (average of the three first measurements before additions). Mean values, for each treatment were calculated as the average of the cycles 5–7. Standard deviations (SD), and 95% confidence intervals of means (CI) are also presented. *N*: number of independent experiments. *Χ*^*2*^ and *p* values were estimated by GLMMs (gamma distribution, inverse link function) using incubation conditions and treatment (baseline, 5 µM oligomycin, 1 µM FCCP, 50 mM 2DOG, 30 mM oxamate, and 1 µM antimycin + 1 µM rotenone) as fixed factors and experiment as a random factor, and likelihood ratio tests. Significant differences (*p* < 0.05) are shown in boldface

OCR showed a reduction of approximately 61% when the cells were treated with oligomycin (Figs. 3A, 4A) and increased to approximately 800% after the addition of FCCP (Figs. 3B, 4A), which is indicative of a high respiratory capacity. In addition, OXPHOS inhibition either with oligomycin (Figs. 3E, 4B) or the combination of rotenone plus antimycin A (Figs. 3F, 4B) resulted in compensatory increases in ECAR. Interestingly, the metabolic response to the glycolytic inhibitors 2DOG and oxamate displayed marked differences. While the hexokinase inhibitor 2DOG caused a decrease in ECAR (Figs. 3G, 4B) and a compensatory increase in OCR (Figs. 3C, 4A), oxamate, a competitive inhibitor of lactate dehydrogenase, caused an inhibition in both OCR (Figs. 3D, 4A) and ECAR (Figs. 3H, 4B).

**Fig. 5:**
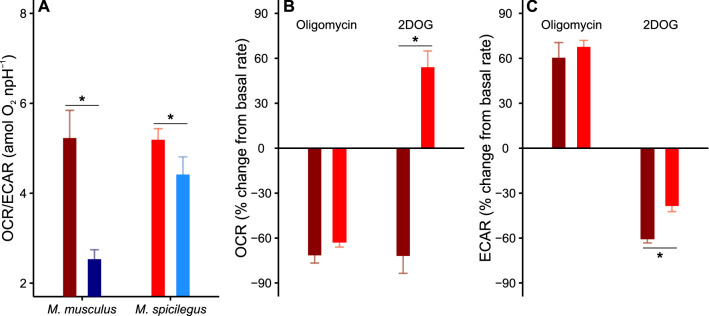
**A** Comparison of the effect of capacitation in the OCR/ECAR ratio between *M. spicilegus* and *M. musculus.* Sperm were incubated for 1 h in non-capacitating (red bars) and capacitating (blue bars) conditions before extracellular flux analysis. **B** Changes in OCR in response to metabolic modulators in non-capacitated sperm of *M. spicilegus* (light red) and *M. musculus* (dark red). Bars on the left: OCR after the addition of 5 µM oligomycin; bars on the right: OCR after the addition of 50 mM 2DOG. **C** Changes in ECAR in response to metabolic modulators in non-capacitated sperm of *M. spicilegus* and *M. musculus*. Bars on the left: ECAR after the addition of 5 µM oligomycin; bars on the right: ECAR after the addition of 50 mM 2DOG. **B**, **C** Values represent the differences relative to the basal rates (0) expressed as percentages. Bars represent means + standard error in all panels. Light bars: *M. spicilegus*; dark bars: *M. musculus*. In (**A**), asterisks indicate significant differences (*p* < 0.05) between incubation conditions for the same species in MMs using incubation conditions and species as fixed factors and experiment as random factor. In (**B**, **C**), asterisks indicate significant differences (*p* < 0.05) between species in MMs using and species as fixed factor and experiment as random factor. OCR/ECAR and the responses of ECAR to metabolic modulators were analyzed using LMMs after log_10_ transformation of the response variable. The responses of OCR to metabolic modulators were analyzed using GLMMs (gamma distribution, inverse link function)

Finally, the relative changes in OCR and ECAR were compared between *M. spicilegus* and a laboratory strain of *M. musculus*. The OCR and ECAR data for *M. musculus* were taken from a recent study by Tourmente et al. [40]. In the two species, capacitation led to qualitatively similar changes in the use of the two energy supply pathways. Both species showed a decrease in basal OCR (*M. musculus*: 37%; *M. spicilegus*: 27%) and an increase in basal ECAR (*M. musculus*: 52%; *M. spicilegus*: 36%). The elevation in ECAR observed in *M. spicilegus* was non-significant, and the change in the OCR/ECAR was more pronounced in *M. musculus* (Table 3, Fig. 5A). It is interesting to note that *M. spicilegus* sperm were less capable of sustaining ATP levels under capacitating conditions despite their enormous respiratory capacity (Fig. 2F).

**Table 3:** Comparison of the effect of capacitation or metabolic modulators on the basal metabolic rates of sperm of two species of the genus *Mus*

Dependent variable	*N*	Species	Non-capacitating			Capacitating			Independent variable	*X*^*2*^	*p*
			Mean	SD	CI	Mean	SD	CI			
OCR/ECAR (amol O_2_ npH^−1^)	11	*M. musculus*	5.23	3.49	1.26	2.53	1.23	0.46	Species^a^	2.64	0.104
									Incubation medium	35.98	** < 0.001**
	6	*M. spicilegus*	5.19	1.75	0.25	4.42	2.55	0.80	Interaction	10.99	** < 0.001**
OCR resp. to oligomycin (%)	4	*M. musculus*	− 72.6	17.9	11.35				Species^b^	0.86	0.354
	4	*M. spicilegus*	− 62.8	11.2	7.13						
OCR resp. to 2DOG (%)	3	*M. musculus*	− 71.2	34.6	26.6				Species^b^	10.18	**0.001**
	3	*M. spicilegus*	53.4	30.5	25.5						
ECAR resp. to oligomycin (%)	4	*M. musculus*	60.4	35.1	22.3				Species^a^	1.09	0.297
	4	*M. spicilegus*	67.6	15.1	9.59						
ECAR resp. to 2DOG (%)	3	*M. musculus*	− 60.0	7.87	6.05				Species^a^	8.71	**0.003**
	3	*M. spicilegus*	− 38.2	9.93	9.19						

*Χ*^*2*^ and *p* values were estimated using LMMs and likelihood ratio tests. The mean responses of OCR to metabolic modulators were calculated as the average across cycles 5–7 for each well. The mean responses of ECAR to metabolic modulators was represented by the highest (oligomycin) and lowest (2DOG) values among cycles 5–7 for each well. Standard deviations (SD), and 95% confidence intervals of means (CI) are also presented. *N*: number of independent experiments. *Χ*^*2*^ and *p* values for the OCR/ECAR analysis were estimated by MMs using species and incubation conditions as fixed factors and experiment as a random factor, and likelihood ratio tests. In the case of the responses of OCR and ECAR to metabolic modulators the MMs used species as fixed factor and experiment as random factor. Significant differences (*p* < 0.05) are shown in boldface

^a^Estimated using LMMs after log_10_ transformation of the response variable

^b^Estimated using GLMMs (gamma distribution, inverse link function)

Striking differences were found between the two species in the response to the inhibition of either OXPHOS or glycolysis. While the inhibition by oligomycin of mitochondrial ATP synthesis in the two mouse species resulted in a compensatory increase in ECAR (Fig. 5B), the inhibition by 2DOG of glycolysis only caused an increase in respiration in *M. spicilegus* (Table 3; Fig. 5B). For *M. musculus*, 2DOG inhibited both respiration and glycolysis (Fig. 5B, C).

## Discussion

The steppe mouse (*M. spicilegus*) has a series of features that make it a relevant model to study murine sperm metabolism. *M. spicilegus* has the highest total sperm number [64], proportion of motile sperm [57, 64], and proportion of capacitated sperm [58, 65] of all *Mus* species examined so far. Its sperm cells also exhibit higher sperm ATP levels [56, 57], faster ATP consumption rate [56], and higher swimming velocity [56, 57, 66] in comparison with other species in this genus. Furthermore, *M. spicilegus* sperm have higher OXPHOS rates and their motility is more dependent on oxidative metabolism than *M. musculus* sperm [39]. These extreme traits are probably the result of intense postcopulatory sexual selection, since in Muroid rodents these sperm parameters are strongly correlated to testes size relative to body mass, a reliable indicator of sperm competition levels [67, 68], and *M. spicilegus* has the largest relative testes size of Palearctic *Mus* species [69, 70]. In addition, *M. spicilegus* belongs to the sister taxon to *M. musculus*, having diverged from their common ancestor approximately 1.2 million years ago [71], and with a genome that shows a high degree of homology with its sister species [72]. All these features define a good model system for the study of diversity of key physiological differences among species and relate them to variations in a very similar genetic background.

Our study revealed significant changes in the bioenergetics of *M. spicilegus* sperm, as a consequence of capacitation, that are consistent with the observations recently published for *M. musculus* [40]. Incubation under capacitating conditions promoted a significant reduction of the OCR/ECAR ratio. This variation is the result of a marked decrease in basal respiration rate, and a slight increase in basal glycolysis. Although the elevation of glycolytic rate was not significant in statistical terms, it is still a relevant result since this change is susceptible to being partially masked by the reduction in respiratory rate [63].

Previous studies concerning the variations imposed by capacitation on mouse sperm have yielded conflicting results despite using similar measuring techniques (EFA). One study [55] found that the rates of glucose uptake, aerobic glycolysis, and OXPHOS increased several-fold when monitored during capacitation time when this process was pharmacologically induced in absence of HCO_3_^−^. These findings are in line with the notion of a general metabolic upregulation in response to a capacitation-induced increase in ATP demand [37, 38, 73]. A different study [40] showed that, after being incubated for 1 h in capacitating conditions (i.e., a medium that contained HCO_3_^−^), sperm exhibited a marked change in the usage ratio of the energy-producing pathways in favor of glycolytic metabolism, without decreasing ATP levels. These results supported the hypothesis that the function of the metabolic changes associated with sperm capacitation would be to sustain local ATP concentrations in the distal flagellar regions instead of attending to increased global ATP demands [46].

In all, the results of the present study suggest that capacitation induces a shift in the usage ratio of the energy-producing pathways in the sperm of *M. spicilegus* toward a less oxidative metabolism, similarly to what Tourmente et al. [40] found in *M. musculus*. These findings are further supported by recent publications showing that, in the sperm of laboratory mice, sperm capacitation occurs concomitantly with hyperpolarization of the mitochondrial membrane [47, 53, 54], which is also necessary for sperm hyperactivation [53, 54]. Since high mitochondrial membrane potential (MMP) is reached when, under physiological conditions, mitochondrial ATP synthesis decreases [74–76], the increased MMP in capacitated sperm [47, 53] would be indicative of lower OXPHOS rates.

Consistently with what was reported in *M. musculus*, the metabolic rates of *M. spicilegus* sperm responded as expected to metabolic modulation, regardless of incubation conditions. Respiratory rate was reduced when sperm were treated with oligomycin or antimycin plus rotenone and elevated when FCCP was added to the medium, while glycolytic rate decreased after the addition of 2DOG or sodium oxamate. Nonetheless, there are several bioenergetic features in which the sperm of these two species differ.

Under non-capacitating conditions, the sperm of *M. spicilegus* swim significantly faster and have more prolonged motility [56, 57] than those of *M. musculus*. This seems to be a consequence of their ability to consume ATP at faster rates [56] while sustaining relatively high ATP concentrations [57]. Moreover, *M. spicilegus* sperm have higher OXPHOS rates and are more dependent on respiration for motility maintenance [39], possibly as a way of sustaining their stringent energetic regime through increased ATP production.

Our findings also support the idea of OXPHOS playing a greater role in sperm bioenergetics in *M. spicilegus*. Whereas sperm capacitation promoted a substantial decrease in respiratory rate in our experiments, the increase in glycolytic rate under these conditions was less pronounced. Furthermore, although ATP consumption rate did not vary between incubation conditions, exposure to capacitating medium resulted in lower ATP concentrations, probably as a consequence of the concomitant decrease in respiratory rate. Previous evidence is consistent with this conclusion since sperm ATP levels have been shown to decrease throughout capacitation in *M. spicilegus* [58], and the changes in sperm motility patterns associated with capacitation in this species (lower progressive velocity and less linear trajectories) [58] are similar to those produced as a consequence of respiratory inhibition [39]. Furthermore, proteomics analyses have found that glycolytic enzymes are significantly less abundant in the principal piece of *M. spicilegus* than in its *M. musculus* counterpart [77]. Finally, analyses of flagellar beating patterns of laboratory mouse sperm under metabolic inhibition suggest that mitochondrial ATP cannot diffuse from the midpiece to the rest of the flagellum at a sufficient rate, making glycolysis the main pathway for local ATP production in the distal portion of the flagellum [46]. Interestingly, since *M. spicilegus* principal piece is 20% shorter than that of *M. musculus* [57], ATP produced in the mitochondria may suffice to supply a larger proportion of ATP to the principal piece.

A striking feature of the metabolism of *M. spicilegus* sperm is their ability to respond to inhibition of one metabolic pathway with an increase in the activity of the other. The sperm of *M. spicilegus* (this study) and *M. musculus* [40] are able to temporarily increase their glycolytic rate in response to OXPHOS inhibition with oligomycin, thus being able to rely on ATP supply by glycolysis when respiration is unavailable. However, the inhibition of the glycolytic pathway using 2DOG promotes a reduction in respiratory rate in *M. musculus* sperm to levels similar to those reached with oligomycin [40, 55], meaning that, in this species, the normal occurrence of OXPHOS depends on endogenous pyruvate sources (i.e., glycolysis in the principal piece). In contrast, the exposure of *M. spicilegus* sperm to 2DOG elicits an increase in OCR in both incubation conditions, indicating that these cells are capable of (a) utilizing exogenous respiratory substrates to fuel OXPHOS in absence of endogenous pyruvate, and (b) perform metabolic compensation toward any of the two main energy production pathways. This represents a higher level of metabolic flexibility that would allow *M. spicilegus* sperm to fully cope with the changing metabolic landscape of the female reproductive tract, which is known to vary between regions [41, 42].

Remarkably, the above-mentioned compensation ability is not exhibited when glycolysis is inhibited with sodium oxamate, a competitive inhibitor of the enzyme lactate dehydrogenase (LDH). LDH is present in mouse sperm as a sperm-specific isoform (LDHC) that is located both in the cytosol and the mitochondrial matrix [78, 79]. Along with a putative lactate carrier [80], this dual LDH disposition allows mouse sperm mitochondria to internalize lactate and reoxidize it to pyruvate, thus transporting reduced equivalents to the mitochondria to fuel OXPHOS while ensuring the progress of glycolysis by the maintenance of the cytosolic of NADH/NAD + ratio [81]. Since, contrary to *M. musculus*, *M. spicilegus* sperm are able to metabolize external respiratory substrates, the inhibition of LDH by oxamate would, at the same time, preclude glycolysis in the principal piece and lactate reoxidation within the mitochondria, effectively starving both metabolic pathways.

Altogether, the results of our study revealed that capacitation elicits changes in the usage ratio of OXPHOS and glycolysis in *M. spicilegus* sperm that are similar in direction to those exhibited by *M. musculus*. Nonetheless, the sperm of *M. spicilegus* presents unique metabolic characteristics, such as a prevalence of respiratory processes over glycolytic ones, and a greater capacity for metabolic compensation, that make this species a relevant model for the study of murine sperm bioenergetics. Furthermore, these outstanding characteristics might shed light on adaptations to selective pressures leading to increased gamete performance in very competitive scenarios.

Finally, these results may pave the way for future studies dealing with sperm metabolic variations and capacitation in human sperm, since these phenomena also appear to be related in this species [82–84]. However, it must be borne in mind that the high degree of phylogenetic relatedness between *M. spicilegus* and *M. musculus* suggests that significant differences in sperm metabolic features may arise quickly through evolutionary time. Thus, given the highly species-specific nature of sperm metabolism, the translation of conclusions between different model species should be taken with utmost caution.

## Supplementary Information

Below is the link to the electronic supplementary material.

Supplementary file1 (PDF 10060 KB)

## Data Availability

The datasets generated during and/or analyzed during the current study are not publicly available but are available from the corresponding author on reasonable request.
