# Supplementary material for: Bioenergetic changes in response to sperm capacitation and two-way metabolic compensation in a new murine model
Source: Cell Mol Life Sci. 2022 Dec 19;80(1):11. doi: 10.1007/s00018-022-04652-0 (PMC9763147; doi:10.1007/s00018-022-04652-0)
Supplement: Supplementary file 1 — Supplementary file1 (PDF 10060 KB) [file 18_2022_4652_MOESM1_ESM.pdf]

Supplementary Information for

Bioenergetic changes in response to sperm capacitation and two-way metabolic compensation in a new murine model

Maximiliano Tourmente[1,2,3], Ester Sansegundo[1], Eduardo Rial[4] and Eduardo R. S. Roldan[1]

[1]Department of Biodiversity and Evolutionary Biology, Museo Nacional de Ciencias Naturales (CSIC), 28006-Madrid, Spain

[2]Centro de Biología Celular y Molecular, Facultad de Ciencias Exactas, Físicas y Naturales. Universidad Nacional de Córdoba (FCEFyN – UNC), X5016GCA Córdoba, Argentina

[3]Instituto de Investigaciones Biológicas y Tecnológicas. Consejo Nacional de Investigaciones Científicas y Técnicas (IIByT – CONICET, UNC), X5016GCA Córdoba, Argentina

[4]Department of Structural and Chemical Biology, Centro de Investigaciones Biológicas Margarita Salas (CSIC), 28040-Madrid, Spain

Supplementary Figure S1. Adhesion of *M. spicilegus* sperm to laminin for extracellular flux assays.

Supplementary Figure S2. Oxygen levels during extracellular flux analysis of *M. spicilegus* sperm.

Supplementary Figure S3. Metabolic parameters for the assessment of sperm bioenergetic phenotype.

Supplementary Table S1. Calculation of metabolic parameters for *M. spicilegus* sperm.

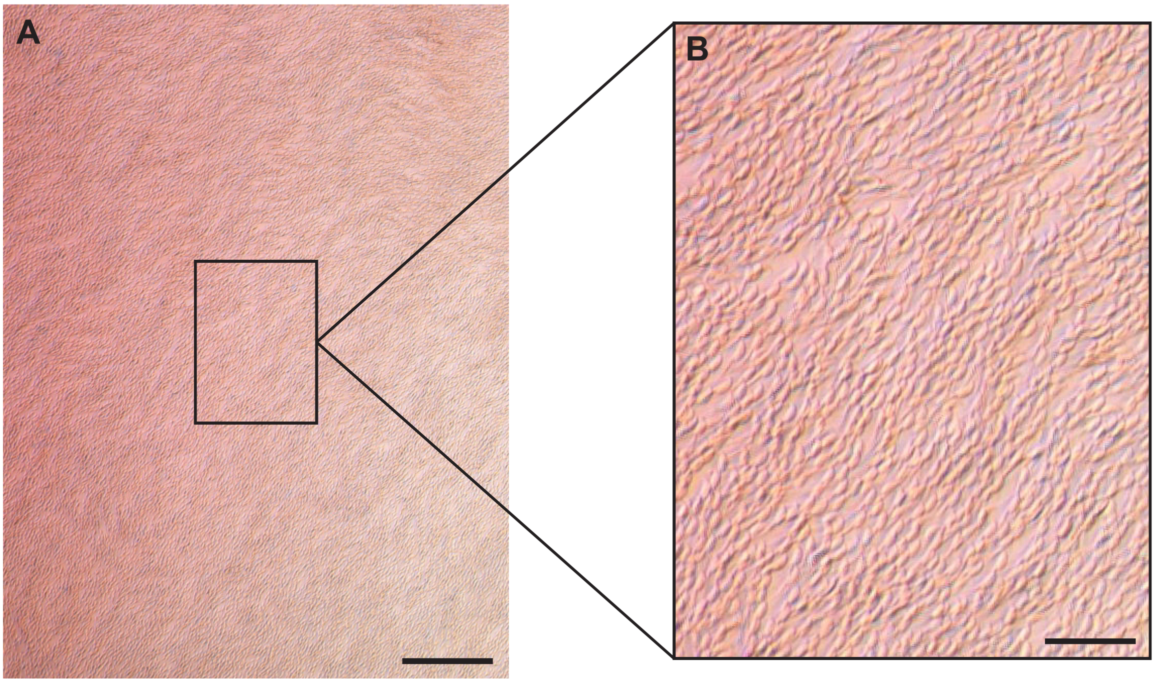

Supplementary Figure S1. Adhesion of *M. spicilegus* sperm to laminin for extracellular flux assays. Full field (A) and zoomed-in (B) micrograph of a representative microscopic field containing *M. spicilegus* sperm adhered to a laminin-coated XF24 microplate. Scale bars: (A) 100 µm, and (B) 25 µm.

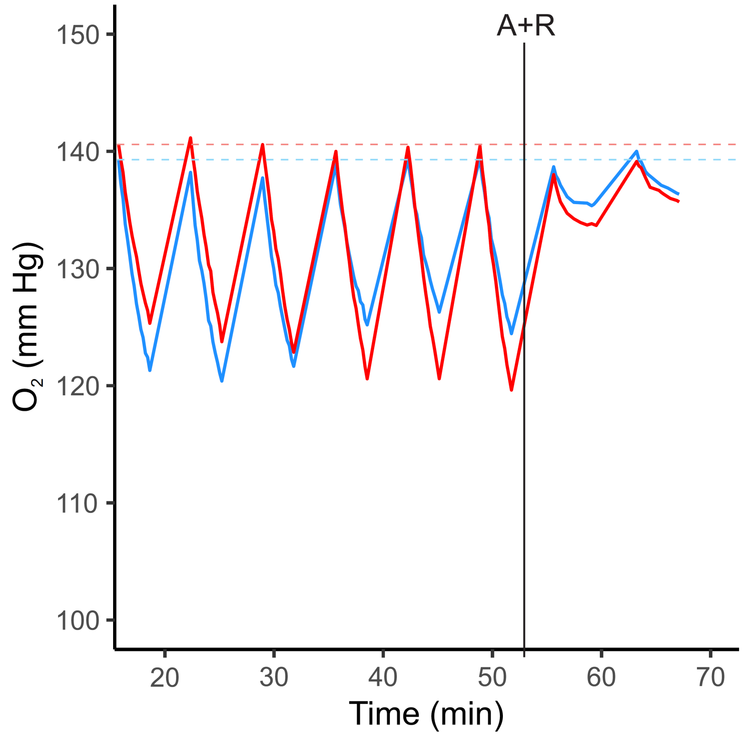

Supplementary Figure S2. Oxygen levels during extracellular flux analysis of *M. spicilegus* sperm. Each oxygen curve corresponds to one well of a Seahorse XF24 microplate throughout a representative experiment. During the decreasing phase, oxygen is consumed by the cell population adhered to the bottom of the plate and the rate of consumption is estimated by the analysis software. After measurement, oxygen levels are recovered by automatic mixing of the medium within the well. Sperm were incubated in non-capacitating (red line) or capacitated (blue line) conditions for 1h previous to the experiment. Dashed horizontal lines: oxygen level at the beginning of the first measurement. Black vertical line: addition of 1 µM antimycin and 1 µM rotenone.

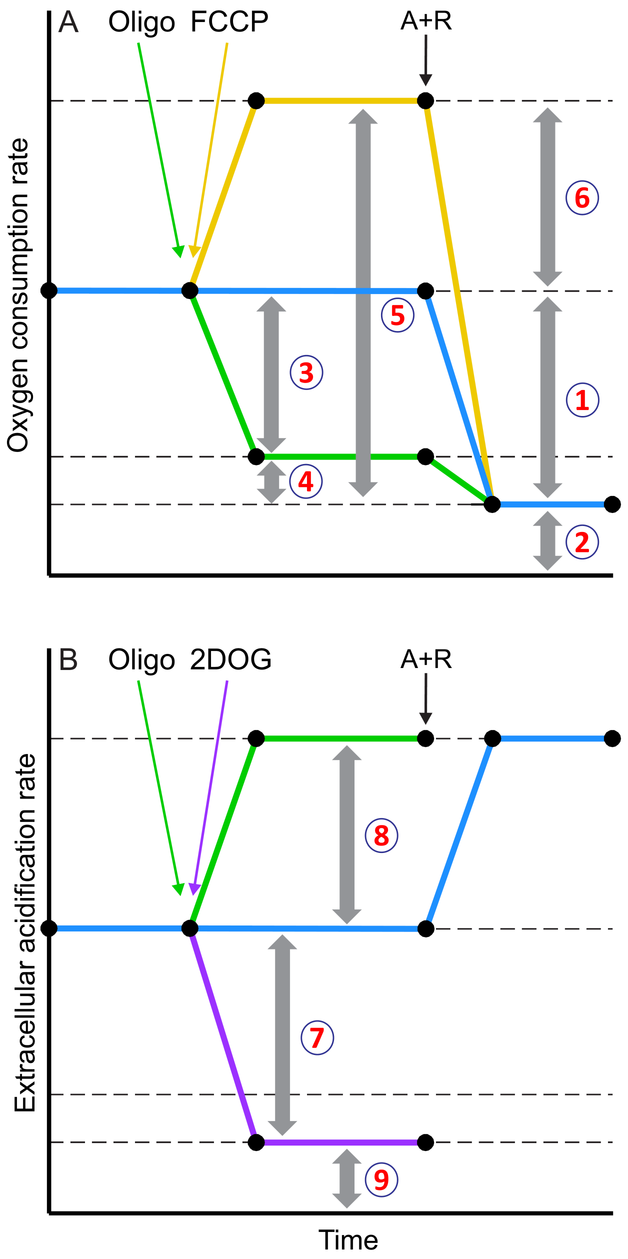

Supplementary Figure S3. Metabolic parameters for the assessment of sperm bioenergetic phenotype. The rates of oxygen consumption (A) and extracellular acidification (B) are monitored while alternative metabolic effectors are added. Oligo: 5 μM oligomycin is added to inhibit mitochondrial ATP synthesis; FCCP: 1 μM FCCP (respiration uncoupler) is added to ensure the maximum rate of respiration is reached; A+R: simultaneous addition of 1 μM antimycin A and 1 μM rotenone to inhibit mitochondrial respiration; 2DOG: addition 50 mM 2-deoxy-glucose to inhibit glycolytic ATP and pyruvate production. The following bioenergetic parameters are calculated for the rates of oxygen consumption and extracellular acidification as indicated in the scheme: 1) Basal respiration rate; 2) Non-mitochondrial oxygen consumption; 3) Respiratory ATP production; 4) Proton leak; 5) Maximum respiration rate; 6) Spare respiratory capacity; 7) Basal glycolysis rate; 8) Glycolytic reserve; 9) Non-glycolytic extracellular acidification.

Supplementary Table S1. Calculation of metabolic parameters for *M. spicilegus* sperm. OCR and ECAR values are obtained from extracellular flux analyses. Each parameter is the result of subtracting the values under condition B from the values under condition A.

Parameter	Condition A	Condition B
Basal respiration rate [a]	OCR before any additions	OCR after the addition of antimycin A and rotenone
Respiratory ATP production [b]	OCR before any additions	OCR after the addition of oligomycin
Proton leak [b]	OCR after the addition of oligomycin	OCR after the addition of antimycin A and rotenone
Maximum respiration rate [c]	OCR after the addition of FCCP	OCR after the addition of antimycin A and rotenone
Spare respiratory capacity [c]	OCR after the addition of FCCP	OCR before any additions
Basal glycolysis [d]	ECAR before any additions	ECAR after the addition of 2DOG
Glycolytic reserve [b]	ECAR after the addition of oligomycin	ECAR before any additions

[a] Cells treated with FCCP were excluded from this calculation. [b] Calculated only in experiments treated with oligomycin and antimycin + rotenone. [c] Calculated only in experiments treated with FCCP. [d] Calculated only in experiments treated with 2DOG.
